# Biochemical and molecular characterisation of *Tetrahymena thermophila *extracellular cysteine proteases

**DOI:** 10.1186/1471-2180-6-19

**Published:** 2006-02-28

**Authors:** Lutz Herrmann, Michael Erkelenz, Ingo Aldag, Arno Tiedtke, Marcus WW Hartmann

**Affiliations:** 1 Cilian AG, Johann-Krane-Weg 42, Muenster D-48149, Germany; 2 Institute for General Zoology and Genetics, University of Muenster, Schlossplatz 5, Muenster D-48149, Germany

## Abstract

**Background:**

Over the last decades molecular biologic techniques have been developed to alter the genome and proteome of *Tetrahymena thermophila *thereby providing the basis for recombinant protein expression including functional human enzymes. The biotechnological potential of *Tetrahymena *has been proved in numerous publications, demonstrating fast growth, high biomass, fermentation in ordinary bacterial/yeast equipment, up-scalability, existence of cheap and chemical defined media. For these reasons *Tetrahymena *offers promising opportunities for the development of a high expression system. Yet optimised high yield strains with protease deficiency such as commonly used in yeast and bacterial systems are not available.

**Results:**

This work presents the molecular identification of predominant proteases secreted into the medium by *Tetrahymena thermophila*. A one-step purification of the proteolytic enzymes is described.

**Conclusion:**

The information provided will allow silencing of protease activity by either knock out methods or by *Tetrahymena *specific antisense-ribosome-techniques. This will facilitate the next step in the advancement of this exciting organism for recombinant protein production.

## Background

*Tetrahymena thermophila *is one of the most extensively examined ciliated protozoa and for decades it has served as a model organism in different research areas. The discovery of telomeres [1] and telomerase [2] as well as RNA-mediated catalysis [3] were discovered and studied in *Tetrahymena*. Within the last decades molecular biological techniques have been developed to alter *T. thermophila*'s genome and proteome: There are DNA transfection methods that allow transformation of either the germline micronucleus (MIC) or the vegetative macronucleus (MAC) [4-6]. Episomal plasmids based on an rDNA-replicon are available [7]. Homologous recombination in either MIC or MAC enable knock-out/-in techniques [8,9]. On protein level heterologous expression of related species has been performed [10,11] and recently the expression of functional human enzymes including proper formation of disulfide bridges and addition of N-glycans has been demonstrated (submitted, BMC Biotechnol). In parallel a few research groups have evaluated the biotechnological potential of *Tetrahymena *within the last years [12-14]. Promising results have been achieved to culture this species with fast growth to high biomasses. Furthermore fermentation processes can be performed in ordinary bacterial/yeast equipment. Additionally up-scalability, one of the most important criteria for industrial production, has been demonstrated successfully. There are also data on *Tetrahymena *continuous high-cell-density fermentation in a perfused bioreactor making this organism even more useful for industrial applications [13]. For all these reasons *Tetrahymena thermophila *has been selected by the US National Human Genome Research Institute as one of the high-priority genomes for sequencing in 2002. Today sequencing of the MAC genome has nearly been completed by The Institute for Genomic Research thus enabling mining of the organism's genome. Tiny amounts of protein, e.g., can be characterized easily by mass spectrometry in connection with a search for obtained peptide fragments within the database. *T. thermophila *secretes many lysosomal enzymes into the surrounding medium [15]: phospholipases [16-18], glycosidases [19], phosphatases [20] and proteases [21] that will modify or even degrade potential product. In a heterologous expression system these undesired enzyme activities must be depressed to assure quality and yield of the product. Today all microbial expression systems can rely on decades of research results including detailed information on the genome and proteome of the used organisms. So, e.g., in *E. coli *systems optimised strains with additional tRNAs and/or protease deficiencies have been engineered and have been available on the market for many years. Yet *Tetrahymena's *commercial potential has not been exploited at all although today all necessary tools for genetic engineering are available. Till now only one protease-sequence for *Tetrahymena *has been identified by experimental means: Tetrain in *T.pyriformis*, a cathepsin L family member [22]. In *T.thermophila *a cDNA encoding for a similar putative protease, pCyp, has been described, but examination of the protein and its expression are lacking [23]. Although Straus *et al*. described the purification of different *T.thermophila *cysteine proteases by conventional chromatography methods they were not able to determine the sequence of the enzymes [21]. As all proteases described so far are cysteine proteases and nearly all proteolytic activity of cell extracts can be inhibited by cysteine protease specific inhibitors we chose a straightforward one-step purification approach described by Greenbaum *et al*. This method makes use of modified trans-epoxysuccinyl-L-leucylamido-(4-guanidino)-butane (E64) and has been adopted successfully for cysteine proteases of different organisms ranging from plants [24] over *P. falciparum *[25] to *D.melanogaster *[26]. Here we provide an additional step in the development of a high performance expression system based on *Tetrahymena *by an in-detail molecular characterization of the major extracellular proteases.

## Results

### Synthesis of DCG-04

**Figure 1:**
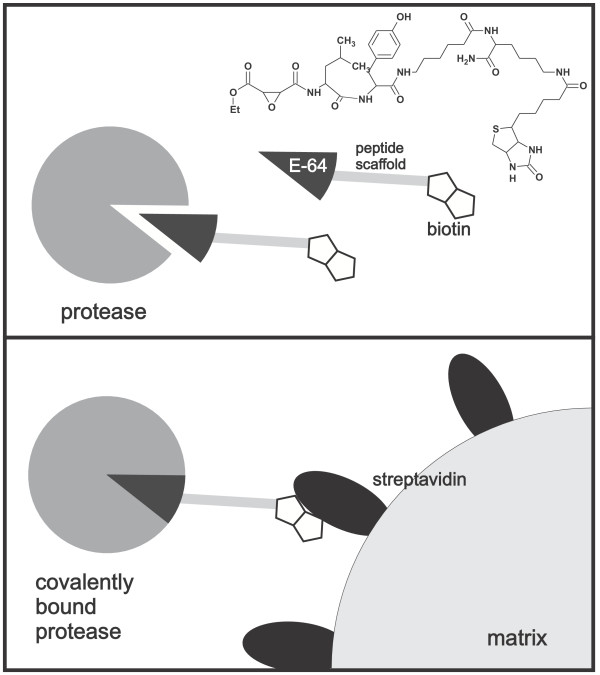
**Purification scheme DCG-04. **The structure of DCG-04 is shown in the upper part. The E64-component binds covalently to active cysteine proteases. Subsequently protease/inhibitor complex is immobilized by a streptavidin bead via the biotin tag. (lower part).

Protease activity profiling is based on labelled protease inhibitors that covalently bind to proteases in an activity dependent manner. These specifically binding reagents can also be used for purification purposes. By linking the cysteine protease inhibitor E64 to biotin Greenbaum *et al*. created a versatile tool called DCG-04for rapid purification of cysteine proteases by immobilizing the substrate/protease complex to streptavidin beads. The method is summarized in figure 1. Synthesis of the substance was performed according to the methods section. The formation of the proper product was verified by mass spectrometry (data not shown).

### Secretion kinetics, production and ex ante characterization of *T. thermophila *extracellular proteases

**Figure 2:**
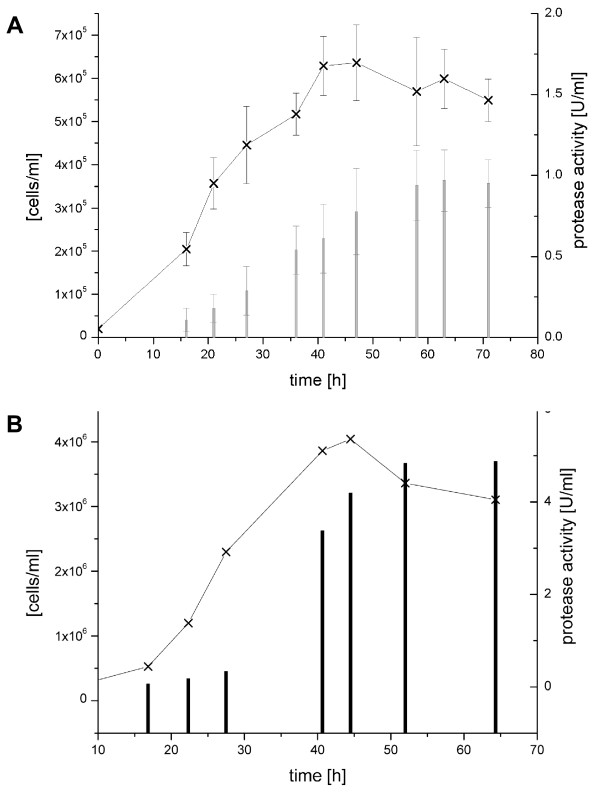
**Growth curve and secretion kinetics of proteases. **A shows the mean cell titers (curve with crosses) and protease activities (bars) during shaker flask cultivation of *T.thermophila *cells. Error bars represent standard errors of three independent experiments. B depicts the cell titers (curve with crosses) and protease activity (bars) during the fermentation used for identification of extracellular proteases are shown.

For production of extracellular proteases in growing *T. thermophila *the secretion kinetics of the enzymes during cultivation in shaker flasks was monitored (figure 2A). Maximum protease activity is observed in late logarithmic growing cell cultures. Consequently to achieve large amount of material, cells were separated from the media of a 2L fermentation process after 65 hours yielding 5 U/ml of protease activity (figure 2B).

**Figure 3:**
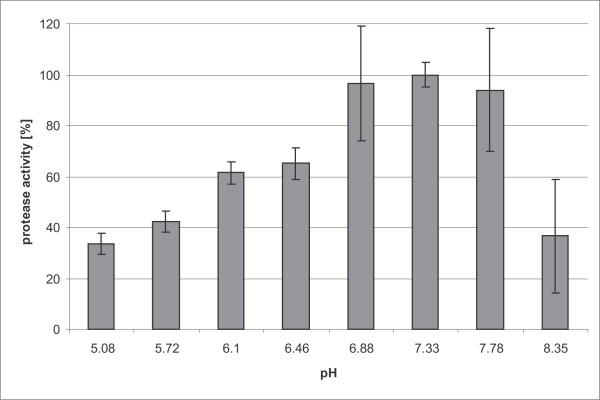
**pH dependency of protease activities. **The pH profile of protease activity in *T. thermophila *supernatants is shown. The overall activity has its maximum at neutral to slightly basic pH. Error bars represent standard errors of three independent experiments.

To evaluate the optimal pH at which the purification would work best the protease activity in the harvested supernatant was determined at different pH values. The results shown in figure 3 suggest optimal conditions at neutral to slight basic pH.

**Figure 4:**
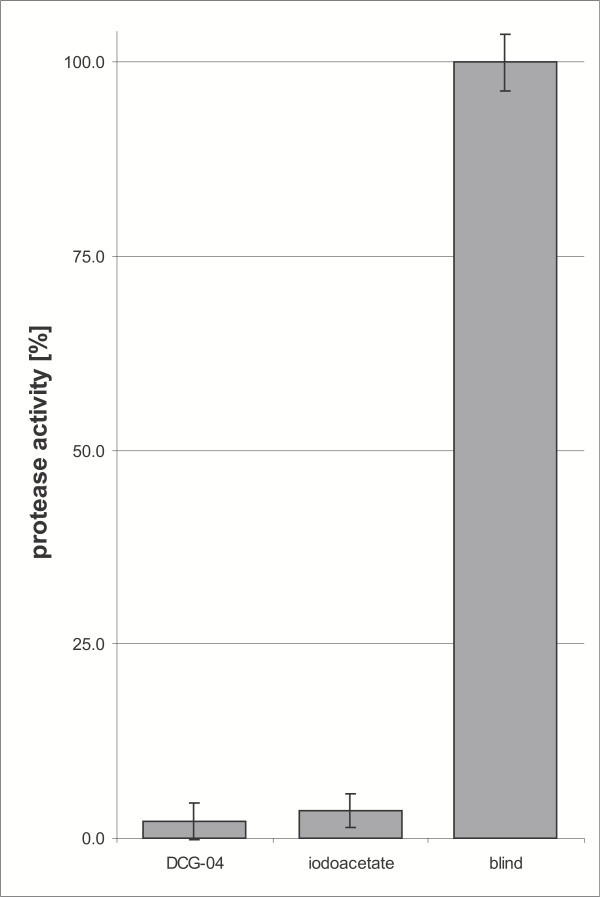
**Inhibition of protease activity by DCG-04. **Nearly all protease activity is inhibited by the addition of DCG-04 (first bar) compared to blind probe (water, very right bar). The inhibition by DCG-04 is comparable to all alkylated enzymes by iodoacetate (middle bar). Error bars represent standard errors of three independent experiments.

To demonstrate that most of the proteolytic activity is due to cysteine and not serine or threonine proteases the inhibitory effect of DCG-04 on the concentrated supernatant was investigated. The strongly alkylating agent lithium iodoacetate served as positive control for enzyme inhibition. Nearly all proteolytic activity vanished by addition of DCG-04 (figure 4); remaining activity is as low as background activity. These findings argue that most of the predominant, secreted proteases are members of the cysteine protease family.

### Purification and identification of *T. thermophila *secreted proteases

**Figure 5:**
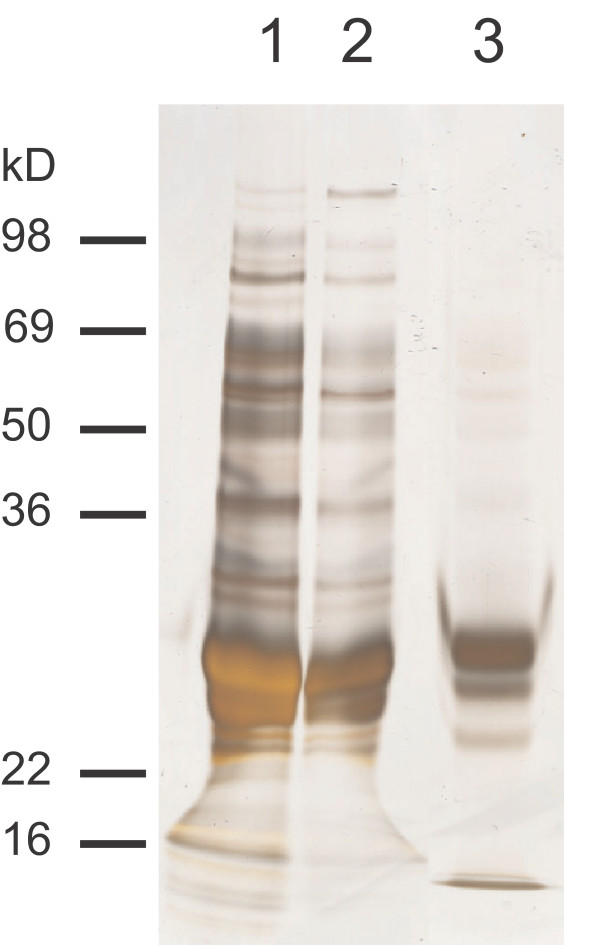
**Purification of DCG-04. **A silver stained SDS-gel of different purification steps by DCG-04. Lane 1 shows the crude *T. thermophila *supernatant, lane 2 the supernatant after the addition of DCG-04 and subsequent extraction by means of streptavidin beads. In lane 3 the supernatant of beads that have been washed and boiled in SDS buffer is shown. The enriched protease fraction mainly consists of bands of approximately 22-28 kD.

**Figure 6:**
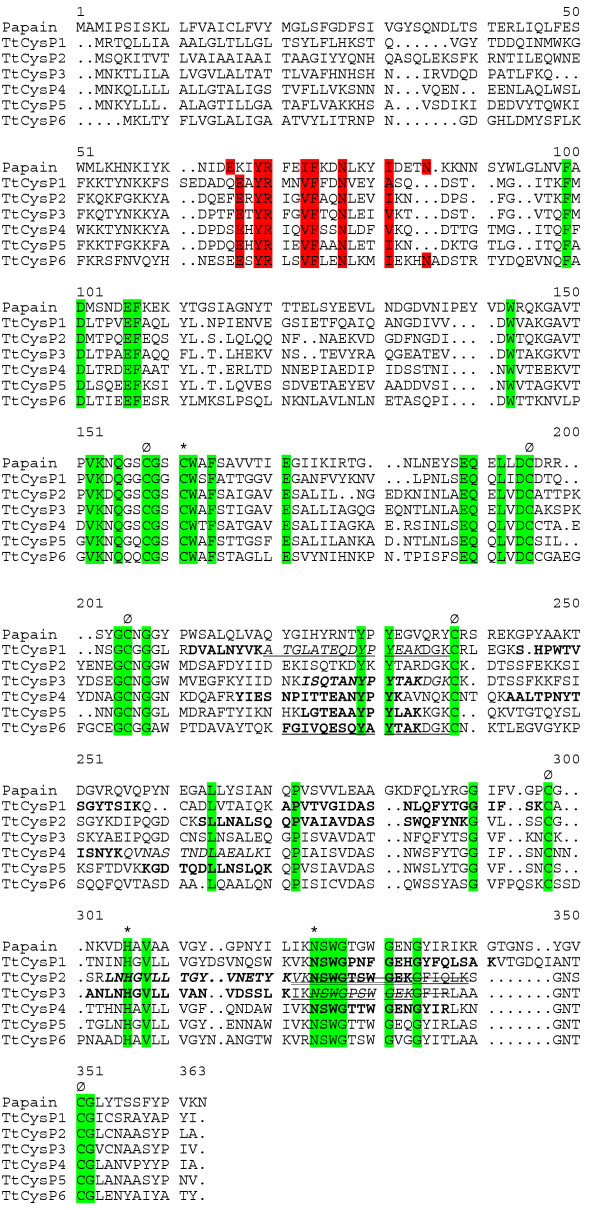
**Sequence alignment of *T. thermophila *extracellular proteases. **The identified cysteine proteases in *T. thermophila *supernatants are compared to papain. The ERFNIN motif is highlighted in red, amino acids that are conserved in all enzymes are green. Amino acids of the catalytic center are marked by *, cysteines that form disulfide bridges by ∅. All peptides found by mass spectrometry in the different proteases are highlighted by either bold, italic, underlined, or doubly underlined letters. Obviously no N-terminal peptides were detected, as only secreted, mature enzymes with cleaved off prepro-peptides (~aa 1-140) were analysed. Peptides around aa 160 are not expected either as the catalytic cysteine that covalently bound to DCG-04 resides at this position.

According to the pH activity profile of secreted proteases of *Tetrahymena *the capturing step with DCG-04 was performed at pH 7.4. Figure 5 illustrates the results of the one-step purification process: The crude supernatants before and after incubation with DCG-04 and streptavidin labelled beads (lane 1 and 2) show a vast and complex band pattern of different sized proteins. The purified protein fraction eluted from the matrix results in predominant bands running at molecular weights ranging from 22 to 28 kD. These sizes have been described for many mature cysteine proteases. To verify the specificity of the purification process, aliquots of the samples were separated by 2-D gel electrophoresis, then blotted to nitrocellulose and finally probed with an anti-biotin antibody. All spots visible on a silver stained reference gel were readily detected by the antibody (data not shown). This argues for an efficient and covalent binding of DCG-04 to the proteases. The bands between 20 and 30 kD were excised and subjected to mass spectrometry. The search algorithm exploited a preliminary database provided by TIGR, a very useful and valuable tool the importance of which has already been predicted in 2000. [27] Six different cysteine proteases of the cathepsin family were unambiguously identified by at least two independent peptides (highlighted in figure 6) termed TtCysP1-6 (*Tetrahymena thermophila *cysteine proteases 1-6). These proteins are listed in the preliminary gene release at The Institute for Genomic Research under temporary identifier 67.m00244, 103.m00134, 103.m00129, 123.m00091, 24.m00272 and 125.m00080 respectively and are located on genomic contig SB210 8254459, SB210 8253891, SB210 8253891, SB210 8254367, SB210 8254649 and SB210 8254370. Whereas the protease pCyp described by Karrer *et al*. [23] was not detectable, sequence alignments reveal that TtCysP6 is *T. thermophila *Tetrain [22] is 72 % identical to the *T.pyriformis *protein. All identified enzymes show a prepro-peptide structure indicated by an ERFNIN motif [23] (figure 6). Signal peptides were predicted by computational analysis (SignalP algorithm [28], table 1). All amino acids essential for enzyme activity are highly conserved (figure 6). As expected no fragments within the first 180 amino acids were found because the prepro-peptide (~140 aa) is cleaved off during the processing of the enzyme. Peptides containing the catalytic cysteine at position ~160 are masked by covalently bound DCG-04.

## Discussion

It is known that the majority of intra- and extracellular proteases in *Tetrahymena *are cysteine proteases. Straus et al. reported, that at least four different proteases are present in *T.thermophila *supernatants. But they were not able to derive any sequences from their data [21]. The only sequence information available on an active protease in growing *Tetrahymena *cells is Tetrain, a *T. pyriformis *derived enzyme. The results presented in this work confirm all data available so far: Chemical targeting of cysteine proteases by means of a mechanism based probe combined with mass spectrometry has allowed identification of six extracellular *T. thermophila *cysteine proteases and their sequences have been determined; the existence of a *T. thermophila *Tetrain has been confirmed by sequence comparison. pCyp, a protease described by Karrer [23], could not be detected in our experiments. A possible explanation is that the cDNA isolates used for detection of pCyp were derived from starved, not from growing cells. This would imply that *Tetrahymena *is able to up- and down regulate different proteases on changing environmental, physiological conditions, a phenomenon that Suzuki *et al*. already postulated for the *T. pyriformis *Tetrain protease [22]. The regulation of various proteins in *Tetrahymena *during different development stages has been reported and investigated in detail by comparing growing, starving and conjugating cells: It was shown that for many differentially expressed proteins the transcriptional activity is the major regulating mechanism[29] This could also be true for pCyp being down-regulated during vegetative growth. Taking a look at the recently acquired genome database of *T.thermophila *one will find far more than 30 different putative genes encoding for cysteine proteases. Table 2 lists the 30 cysteine proteases that are most similar to TtCysP1. All of them have a functional signal peptide according to SignalP analysis and the conserved amino acids of the ERFNIN motif are also present in nearly any putative enzyme. These findings argue that *T.thermophila *is able to choose in a regulated way from a whole set of different proteolytic enzymes, that must be secreted. Further experiments to verify this hypothesis need to be done.

## Conclusion

The main aim in this study was to identify most active proteases in growing ciliate cells as this is the phase ideally suited for expression of foreign genes. Well established *E. coli *and yeast based expression systems have been making use of protease deficient strains for decades to enlarge their product yields. The information provided by the above results therefore is urgently needed for genetic engineering in strain optimisation. To develop a competitive, alternative expression platform based on *T. thermophila *the identified proteases must selectively be knocked out.

## Methods

### Synthesis of DCG-04

The active inhibitory epoxide group ethyl-(2S,3S)-oxirane-2,3-dicarboxylate was generated by adding 5.25 ml 0.1 M KOH in EtOH drop wise to 100 mg diethyl-(2S,3S)-(+)-2,3-epoxysuccinate (Acros Organics) in 3 ml EtOH on ice within two hours and further stirring at room temperature for one hour. A white precipitate formed upon standing at -20°C overnight which was recovered by evaporating EtOH. The residue was taken up by 4 ml 50 mM NaHCO_3 _(aq) and extracted twice with 4 ml EtOAc. The aqueous phase was shifted to pH 2.5 by addition of 2 M KHSO_4 _and was extracted five times with 3 ml EtOAc. Combined organics were dried and concentrated yielding a greenish oil which solidified upon further standing. Proper formation of product was confirmed by mass-spectrometry. Solid phase synthesis of DCG-04 was performed according to a modified protocol published by Greenbaum *et al*. Resin and amino acids were purchased from Nova Biochem. All procedures were carried out under Argon atmosphere at room temperature. Each Fmoc deprotection was carried out by two incubations (1 min and 30 min each) of 20 % piperidine in DMF. During coupling equimolar concentrated solutions of 1-hydroxybenzotriazole, N, N'-diisopropylcarbodiimide and amino acid in DMF were used. Thorough washing with DMF and CH_2_Cl_2 _was performed between each synthesis step. 288 mg of rink amide AM resin were swollen in DMF and deprotected. Fmoc-Lys(biotin)-OH (190 mg, 1.5 eq., 2 h), Fmoc-6-aminohexanoic acid (Fluka, 226 mg, 3 eq., 1 h), Fmoc-Tyr(tBu)-OH (460 mg, 5 eq., 1 h), Fmoc-Leu-OH (353 mg, 5 eq., 1 h) and ethyl-(2S, 3S)-oxirane-2,3-dicarboxylate (68 mg, 2 eq., 1 h) were coupled successively at indicated molar excess. The peptide was cleaved off the resin by addition of cleavage buffer (95 % TFA, 2.5% H_2_O, 2.5 % triisopropylsilane) and precipitated as a white solid by ether at 0°C. The crude peptide was analysed by mass-spectrometry and used in subsequent experiments without further purification.

### Cultivation of *Tetrahymena*

The *T. thermophila *strain CU438 was cultivated in a Bioengineering Kleinlaborfermenter on modified medium as described previously. Cell free supernatants were concentrated ten fold.

### Protease activity assay

Protease activity was determined on microtiter plates by the substrate N-benzoyl-DL-arginine p-Nitroanilide (BAPNA): 50 μl sample were mixed with 200 μl buffer TED (200 mM Tris, 2 mM DTT, pH 7.5) and 10 μl BAPNA solution (20 mg/ml). A kinetic curve of the optical density at 410 nm was tracked on a microtiter plate reader for one hour. Papain (3.3 U/ml) served as reference and activity was calculated by linear regression of the recorded slope.

### Purification of extracellular proteases

Cell free supernatants of *Tetrahymena *fermentation media were adjusted to 50 mM Tris pH 7.4 and 5 mM DTT and incubated with a final concentration of 0.2 mM DCG-04 for 2 h at room temperature. The samples were dialyzed against buffer B (50 mM Tris, 150 mM NaCl, pH 7.4). SDS was added to a final concentration of 0.5 % and samples were boiled for 10 min. Subsequently the samples were diluted with buffer B until the SDS concentration was as low as 0.2 % followed by shaking with pre-equilibrated streptavidin beads (Molecular Probes) at room temperature for one hour. The beads were thoroughly washed with buffer B, boiled in Laemmli-sample buffer and supernatants were subjected to SDS-PAGE. Protein bands were stained with Coomassie or silver.

### Sequence determination of proteases

Protein bands of interest were cut out of the gel and tryptic in gel digestion was performed according to standard protocols. Samples were analysed on a set-up consisting of a Dionex Ultimate HPLC equipped in combination with a Famos autosampler, a Switchos switcher and a ThermoElectron LTQ mass spectrometer. Peptides were identified using Sequest software fed with *T. thermophila *preliminary protein sequence data, that was obtained from The Institute for Genomic Research website. [30] Sequence alignments were performed with Multalin version 5.4.1 [31] (Symbol comparison table: blosum62, Gap weight: 12, Gap length weight: 2, Consensus levels: high = 90% low = 50%).

## Authors' contributions

LH synthesized DCG-04, participated in project conception and drafted the manuscript. ME carried out the purification, SDS gels and enzyme assays and evaluated MS and sequencing data. IA developed protease assays and supervised fermentation processes. AT helped to draft the manuscript. MWWH conceived of the study and participated in its design and coordination. All authors read and approved the final manuscript.

## Figures and Tables

**Table 1:** Signal peptide prediction by SignalP analysis

Protease	TIGR temporary identifier	Signal peptide propability [%]	Max cleavage site probability [%]	Behind amino acid
TtCyp1	67.m00244	93	27	19
TtCyp2	103.m00134	98	42	21
TtCyp3	103.m00129	99	77	22
TtCyp4	123.m00091	91	38	22
TtCyp5	24.m00272	99	78	19
TtCyp6	125.m00080	89	30	20

**Table 2:** Putative cysteine proteases in the *T. thermophila *genome

**Name**	**SignalP**	**ERFNIN motif**								
	**propability [%]**	**E**	**X**_2/3_	**R**	**(I/V)**	**F**	**N**	**(I/V)**	**X**_1/2/3_	**N**
136.m00075	79	E	3	R	V	F	N	-	-	-
295.m00026	73	E	2	R	V	F	N	I	2	N
295.m00023	98	E	2	R	I	F	N	V	2	N
1.m00874	97	E	3	R	V	F	N	I	-	-
103.m00130	93	E	2	R	V	F	N	I	-	-
5.m00542	98	E	2	R	V	F	N	V	1	N
24.m00271	99	E	2	R	I	F	N	I	-	-
5.m00535	98	E	2	R	V	F	N	V	-	-
147.m00097	98	E	2	R	V	F	N	I	-	-
120.m00105	99	E	2	R	V	F	N	V	1	N
150.m00098	96	E	3	R	I	F	N	I	3	N
89.m00111	99	E	3	R	V	F	N	I	3	N
304.m00023	100	-	-	R	I	F	N	I	3	N
13.m00464	93	-	-	R	I	F	N	V	3	N
61.m00268	96	-	-	R	I	F	N	I	-	-
9.m00484	100	E	3	R	V	F	N	V	3	N
9.m00485	100	E	3	R	V	F	N	I	3	N
9.m00486	100	-	-	R	V	F	N	I	3	N
98.m00123	98	E	3	R	V	F	N	V	3	N
98.m00125	98	E	3	R	V	F	N	V	3	N
98.m00126	98	E	3	R	V	F	N	V	3	N
98.m00131	100	E	3	R	V	F	N	V	3	N
98.m00128	98	E	3	R	V	F	N	V	3	N
156.m00128	100	E	3	R	V	F	N	I	3	N
156.m00129	100	E	3	R	V	F	N	I	3	N
343.m00015	100	E	3	R	V	F	N	I	3	N
343.m00014	100	E	3	R	V	F	N	I	3	N
156.m00109	100	E	3	R	V	F	N	I	3	N
5.m00554	100	E	3	R	I	F	N	V	3	N
49.m00253	100	E	3	R	V	F	N	I	-	-
218.m00064	100	E	3	R	L	F	N	I	3	N
